# In situ laser fenestration for revascularization of aortic arch during treatment for iatrogenic type A aortic dissection

**DOI:** 10.1186/s13019-019-0877-z

**Published:** 2019-05-02

**Authors:** Minjian Kong, Jianfang Qian, Qunjun Duan, Xuebiao Li, Aiqiang Dong

**Affiliations:** Department of Cardiovascular Surgery, Second Affiliated Hospital, School of Medicine, Zhejiang University, No.88 Jiefang Road, Hangzhou, 310009 People’s Republic of China

**Keywords:** Iatrogenic aortic dissection, Endovascular stent-graft implantation, Laser in situ fenestration

## Abstract

**Background:**

Iatrogenic aortic dissection is a rare and fatal complication. Its treatment was challenging and controversial especially in patients with previous cardiac procedure. This study aimed to present the case of a patient with aortic dissection after previous open cardiac surgery who was successfully treated by in situ laser fenestration for revascularization of aortic arch.

**Case presentation:**

A 65-year-old man suffered severe aortic and mitral valve regurgitation was treated by open cardiac aortic valve replacement (biological valve, Edwards) and mitral valve repair. During the sixth-month follow-up, computed tomography angiography (CTA) scan revealed an aortic dissection that extended from the ascending aorta to both femoral arteries. After stabilized by medical treatment, the patient was treated by endovascular stent-graft implantation and in situ laser (holmium laser, energy: 0 5 J, frequency: 5 Hz.) fenestration for revascularization of aortic arch in our one-stop hybrid operating room. The patient recovered without any clinical complication and was discharged 5 days after the procedure.

**Conclusions:**

Our work suggested that in situ laser fenestration for revascularization of aortic arch is a feasible, effective, and safe treatment in patients with iatrogenic aortic dissection.

## Introduction

Iatrogenic aortic dissection (IAD) is a rare and fatal complication (≈0.06%) [1]. It can occur after open cardiac surgery, percutaneous coronary intervention (PCI), endovascular aortic aneurysm repair, and transaortic valve replacement. Treatment of IAD in patients with previous cardiac procedure (PCS) remains challenging and controversial. Hence, conventional open and endovascular aortic repair have been advocated. This study aimed to present the case of a patient with IAD after previous open aortic valve replacement, who was successfully treated with total endovascular stent-graft implantation and in situ laser fenestration for revascularization of aortic arch.

## Case presentation

**Fig. 1:**
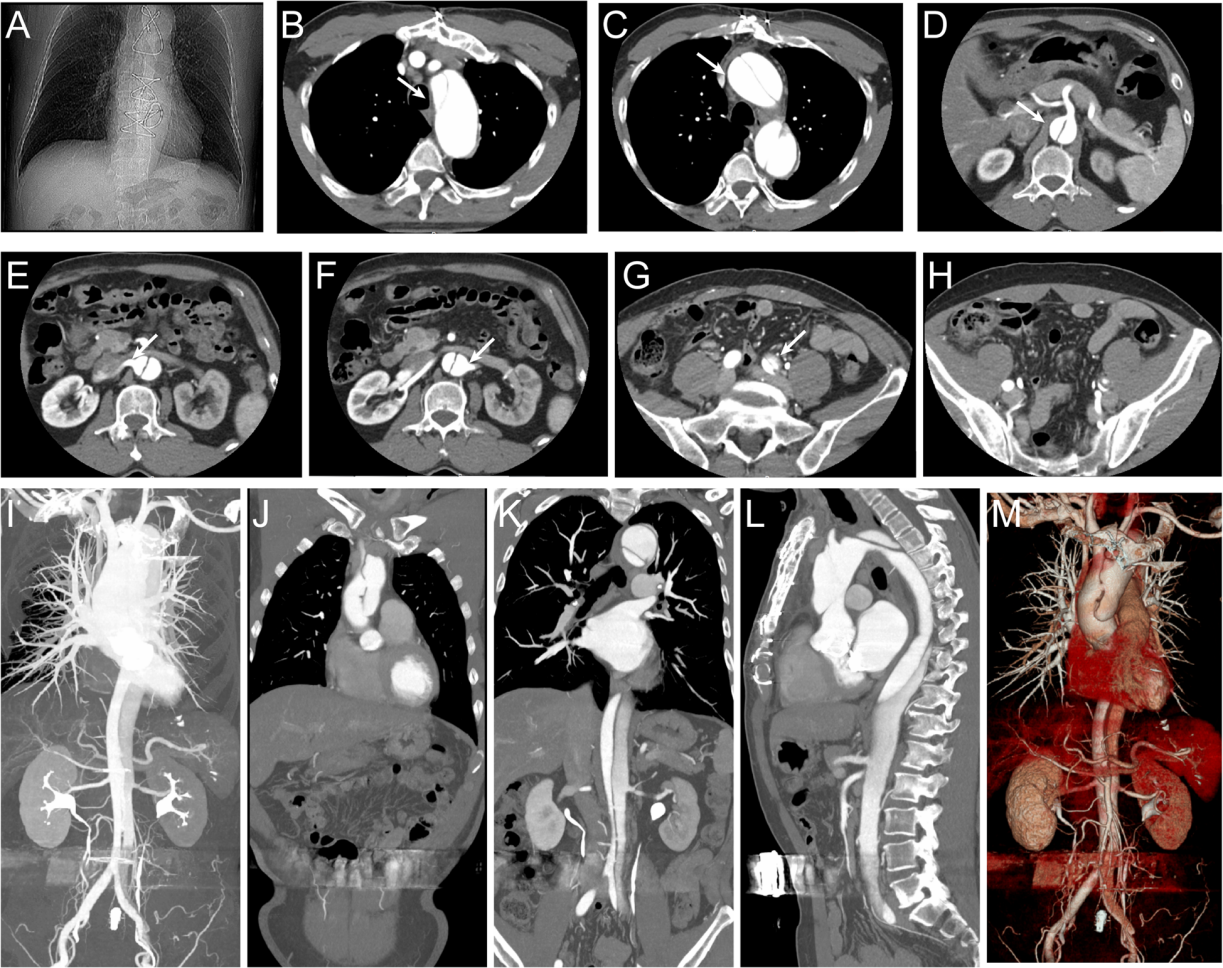
The iatrogenic ascending aorta dissection was identified by computed tomographic angiography (CTA) scan at the sixth month fellow up after aortic valve replacement (the arrow). (**a**-**c**) The site of dissection was about 4 cm above coronary ostium extending distally to iliac artery; (**d**-**g**) Blood supplying of superior mesenteric artery, right renal artery was from true lumen; (**h**-**m**) Blood supplying of left renal artery was from false lumen, and blood supplying of celiac artery was from true and false lumen

A 65-year-old man suffered from chest tightness was admitted to our center seven months ago. Echography results demonstrated severe aortic and mitral valve regurgitation. He was treated with open cardiac aortic valve replacement (biological valve, 25#, Edwards) and mitral valve repair. During the sixth-month follow-up after cardiac procedure, chest radiographs revealed suspected intimal patches of aortic arch and descending aorta, further, thoracic aortic angiography showed aortic dissecting aneurysm (Fig. 1). The site of dissection was about 4 cm above the coronary ostium, where the aortic cannula placed, indicating that it might be caused by inappropriate string technique in surgery. The patient was presented with no chest pain, tightness, syncope, nausea or vomiting. After conservative medical treatment for one month, he came to our department and received endovascular stent-graft implantation and in situ laser fenestration for revascularization of aortic arch.

**Fig. 2:**
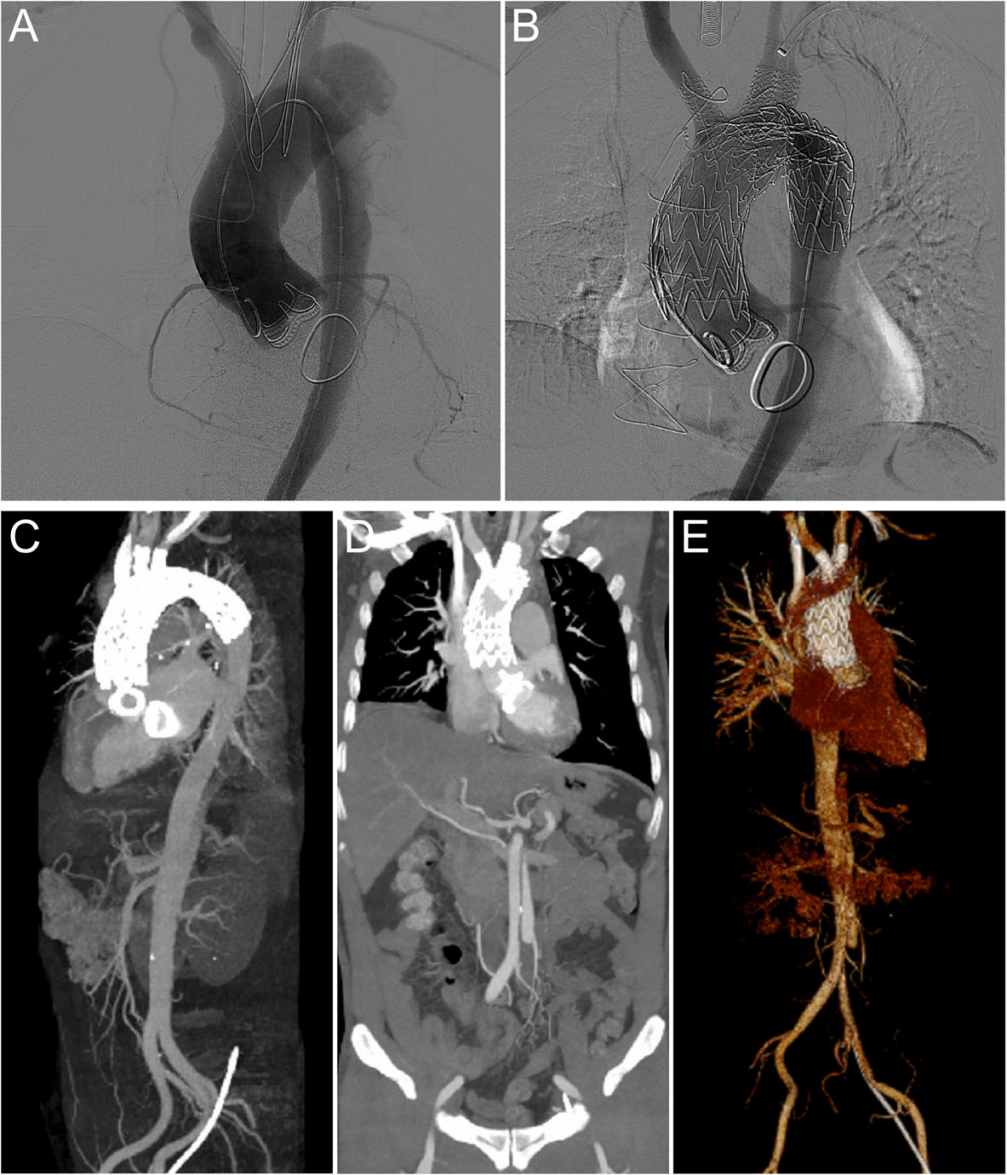
In situ laser fenestration for revascularization of aortic arch during treatment for iatrogenic type A aortic dissection. (**a**) Aortography was performed via the left femoral artery to measure the diameters of the ascending aorta and arch vessels; (**b**) Aortography was performed after stent implantation demonstrated no endoleak, and aneurysm and dissection; (**c**-**e**) CTA presented possible access sites of fenestration, stent placement, patency of the aortic arch branches. CTA, computed tomographic angiography

Femoral vein-bilateral carotid bypass was established by femoral vein (20 F catheter sheath) and bilateral carotid (12–16 F catheter sheath) cannulation. The stent release system (40–40*200 mm, Gore, USA) was introduced. The anchorage area was located 1 cm above the coronary artery orifice, and the stent was released after the systolic blood pressure was reduced to 90 mmHg. The laser catheter was introduced through the left carotid artery, directly contacting the endograft membrane as perpendicularly as possible. Fenestration was made by applying 0 5 J energy (holmium laser, frequency: 5 Hz.), followed by 4-mm balloon dilation. Then, a 0.035-in. stiff guidewire was selected and advanced into the endograft lumen to introduce a bare stent (10 × 38 mm^2^).The same procedure was performed for the left carotid artery and left subclavian artery (Fig. 2). The operation was completed within 4 h, and the time of extracorporeal circulation was 56 min. The patient recovered without any clinical complications and was discharged five days after the procedure.

## Discussion

Type A aortic dissection is an aortic disease associated with high morbidity and mortality [1, 2]. It can occur after cardiac procedures at sites of mechanical trauma owing to preexisting aortic wall pathology. Treatment for patients with previous cardiac procedure (PCS) bears higher risk. Also, the data on the clinical management are limited. In the last decade, total arch replacement combined with stented elephant trunk implantation remained a “standard” therapy [2]. However, even in centers of excellence, this procedure was associated with increased chances of mortality and major cerebrovascular disorders. Owing to the development of the catheter technique, endovascular treatment has developed as a useful tool for selected patients with Type A aortic dissection (TAAD) [3]. A branched and fenestrated endograft seems to be the most promising approach. However, waiting for designed devices is time-consuming, hindering its applicability. In situ fenestration is another potential technique for arch vessel revascularization. Penetration can be performed in many different ways: a needle sharp end of a guidewire, a radiofrequency probe, or laser-generated fenestration [4]. The in situ fenestration technique was used in this case because the laser could provide rapid in situ fenestration of the endograft membrane and the laser fiber was soft and adapted to complex aortic arch anatomy easily [5]. From November 2016 to February 2017, 12 consecutive patients with aortic disease were treated with holmium laser in situ fenestration to revascularize the branches of the superior aortic arch in our center. The success rate of operation was 100%. No type I or II endoleak occurred. One case had left branch retinal artery embolism after operation. Our work indicated that in situ laser fenestration is a feasible, effective, and safe treatment method to revascularize the aortic arch branches, thereby showing great potential for clinical applications. However, the robustness of this technique needs to be verified through long-term follow up in a larger patient population.

## Conclusions

In patients with a very high operative risk, in situ laser fenestration for revascularization of aortic arch is a feasible, effective, and safe treatment.

## Abbreviations

CTA: Computed tomography angiography
IAD: Iatrogenic aortic dissection
PCI: Percutaneous coronary intervention
PCS: Previous cardiac procedure
TAAD: Type A aortic dissection
